# Psychosynthesis: A Foundational Bridge Between Psychology and Spirituality

**DOI:** 10.1007/s11089-017-0753-5

**Published:** 2017-01-27

**Authors:** Catherine Ann Lombard

**Affiliations:** 2 University of Twente, P.O. Box 217, 7500 AE Enschede, The Netherlands; 1 Istituto di Psicosintesi, Centro di Firenze, Via S. Domenico, 16, 50133 Florence, Italy

**Keywords:** Spirituality, Psychosynthesis, Religion, Psychological model, Pastoral care

## Abstract

Pastoral psychologists have long tried to establish a working model that encompasses the seemingly conflicting disciplines of science and religion. Psychosynthesis, a transpersonal psychology and therapeutic approach, offers such a model of the human personality, in which the psychological and spiritual perspectives can converge. This article explores psychosynthesis psychology and therapy as a theoretical framework for pastoral psychology. Although psychosynthesis psychotherapy relies on an array of techniques, it fundamentally works with the clients’ will while emphasizing, exploring, and cultivating their relationships on all levels—intrapersonal, interpersonal, and with the Higher Self. In addition to the subconscious, psychosynthesis includes a higher psychological plane, called the superconscious, from which our higher ethical, aesthetic, scientific, and spiritual values are derived. This article begins by introducing psychosynthesis concepts and techniques. It then provides qualitative findings showing that psychosynthesis counseling helped to awaken spirituality in three out of eleven clients who had formerly identified themselves as atheists. In addition, testimonies are included that show that psychosynthesis counseling also helped all eleven clients to attain personal growth. Finally, the counselor describes her experience of psychosynthesis as a Christian in the therapeutic setting. The framework of psychosynthesis psychology and its techniques are viable methodologies for anyone searching to incorporate spiritual growth into a psychological working model.

Forty-one years have passed since Haronian (1976) offered the readers of this journal his personal overview as a psychotherapist of the rich capacity of psychosynthesis to bridge the chasm between psychology and religion—yet his call remains unheeded. Perhaps more than ever before, pastoral psychologists are struggling to fit religious, spiritual, and transcendent experiences into a scientific model of the human psyche that includes unquantifiable qualities such as forgiveness, patience, good will, and courage (Slife and Richardson 2014). This struggle seems to revolve around the seeming conflict between logical thought and a richer, multidimensional system of analogical thought. The aim of this article is to reintroduce psychosynthesis psychology in the belief that it offers a psychological perspective on spiritual experience that can be put into service of the Christian faith. Psychosynthesis provides an open model of the human psyche that includes a Higher Self along with working concepts that include the human drive towards personal and spiritual growth, the basic need for relationship, various aspects of will—including a transpersonal will—and the individual’s interconnectedness to all in the universe. In addition to providing an overview of psychosynthesis psychology, this article includes testimonies from qualitative research conducted with eleven scientists who received psychosynthesis counseling, three of whom, as self-identified atheists, exhibited spiritual growth. Finally, based on the theoretical introduction, the research findings, and my own personal experience as a Christian psychologist and counselor, I will discuss how the perspectives of psychology and spirituality converge within the psychosynthesis context.

## An overview of psychosynthesis psychology

Psychosynthesis psychology was developed by Roberto Assagioli (1888–1974), who was a medical doctor, contemporary of Freud and Jung, and the first psychoanalyst in Italy. In the 1960s, along with Maslow, he was one of the founders of the emerging field of transpersonal psychology, helping to define essential transpersonal concepts such as peak experiences (Maslow 1968) and the transpersonal or Higher Self (Assagioli 2000). The following is a brief overview of psychosynthesis concepts, including Assagioli’s model of the human psyche, the processes of personal and spiritual psychosynthesis, the will and its four aspects, the role of relationship in personal and spiritual growth, and the intrinsic unity that holds all the diversity around us.

### Assagioli’s model of the human psyche

**Fig. 1:**
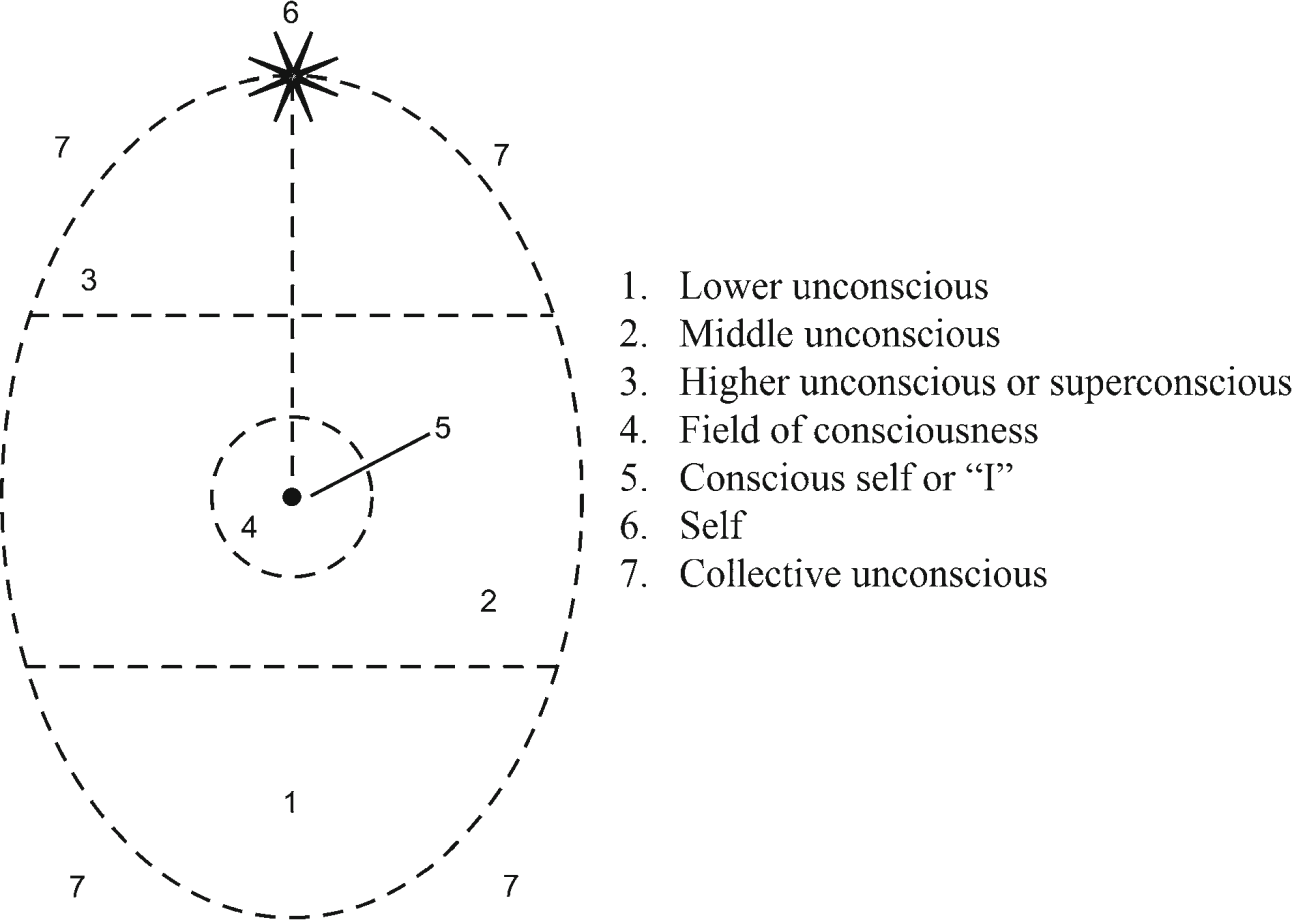
Assagioli’s (2000, p. 15) model of the structure of the psyche. Note: Dotted lines indicate permeable boundaries

In 1933, Assagioli (2000) published in English his model of the human psyche (Fig. 1), which he described as a “conception of the constitution of the human being in his living concrete reality” (p. 14). As I understand Assagioli, he was not interested in creating a doctrine from his ideas but preferred a system that would remain open to possibilities of growth and change.

Perhaps of most interest to the pastoral psychologist is the understanding that the human psyche includes a higher dimension of transpersonal consciousness or superconsciousness as well as a fundamental connection and relationship between the Higher Self and the personal “I.” This relationship is perhaps the most important concept in which the Christian and psychosynthesis perspectives converge. Assagioli (2000) defined the Higher Self (or, more simply, the Self) as a “*unifying and controlling Principle of our life*” (p. 21, emphasis in original). The Self, represented in Fig. 1 as a star, appears as *6* at the top of the diagram. Paradoxically, the Self is both personal and universal—it is dual in nature. Assagioli (2000) also maintains that in addition to consciousness, the Self has transpersonal will, which is “an expression of the Self, and its action is felt by the personal self as a ‘pull’ or ‘call’” (p. 113). In this way, the Self often compels the human personality to express higher qualities such as beauty and compassion as well as to perform actions that involve sacrifice, hardship, risk, and courage. Haronian (1976) explained the Self as

an inner source of wisdom, altruism, growth, and creativity, which, when we are able to reach it, is a better source of guidance and direction for us than either the advice of others or the decision at which we arrive in a more intellectual way. (p. 21)

Assagioli (2000, p. 19) stated that the Self is “above, and unaffected by, the flow of the mind-stream or by bodily conditions,” but rather is a mystic state, a reality that has been testified to by a vast number of individuals throughout history and across cultures. Consequently, Assagioli asserted that the Self is not necessarily religious, dogmatic, or tied to any specific symbolic forms.

With the Self incorporated into his model, one could assert that the Self, as defined by Assagioli, has a more systematic role than the Self as defined by Jung. The Self as proposed by Jung (1979) is a fundamental transcendent archetype that expresses human wholeness and the union of opposites, most generally the union of the polarity of the conscious and unconscious. Jung’s concept is strikingly different from Assagioli’s concept of the Self in that, according to Jung, the Self (like all archetypes) cannot be directly experienced by the individual but is rather a guide and attractor through the process of individuation. In contrast, Assagioli believed that the Self is a reality that can be directly experienced by the individual, and is actually the key part of the individual (as opposed to outside the person) that connects the transpersonal with the personal and, hence, the personal with the universal. For a full description of the similarities and differences between Assagioli and Jung, please refer to Rosselli and Vanni (2014).

This connection of the Self with the personal self or “I” (*5*) is shown in Fig. 1 as a dotted line. The “I” is the center of pure consciousness and will. One can think of the “I” as a pale reflection of the full potential of the Self, and this connection between the “I” and the Self is referred to as the I–Self connection. Like the Self, the “I” is permanent and unchangeable; it is the “inner still point that we experience as truly ourselves” (Hardy 1987, p. 28). Unlike the ego in Freudian thinking, the “I” is exclusively the observer and director of the individual’s actions, not the functions or roles of the personality nor a mediator between demands of the id, superego, and outer world (Haronian 1976). What distinguishes psychosynthesis from most other psychologies is the understanding that the Self relates to the higher qualities within human beings that allow them to foster their I–Self connection and grow towards their authentic personality or “I.” One’s authentic personality is defined as an “expression of the natural, authentic sense of who we truly are,” which is “more than the sum of one’s social roles” (Firman and Gila 2002, p. 48). The “I” is situated within the personal field of consciousness (*4*). This field of personal consciousness is our everyday reality—the limited awareness that we have of ourselves at any particular moment, that is, “the sensations, images, thought, feelings, and impulses which we can observe, analyze and judge” (Assagioli 2000, pp. 15–16). With psychosynthesis, one attempts to expand this field of consciousness by processing unconscious and superconscious material as it arises in the here-and-now.

### The different levels of personal unconsciousness

Assagioli assumes that most people live within a relatively small field of consciousness, its size dependent on the individual’s personal awareness. Nevertheless, no matter how aware we might be, we also live within our fields of personal unconsciousness, which Assagioli has subdivided into lower (*1*), middle (*2*), and higher (*3*) unconsciousness (see Fig. 1). In addition, all unconscious material is interfacing with what Jung (1971, pp. 59–69) called the “collective unconscious” (*7*). In accordance with the Freudian concept of the “unconscious,” the lower unconscious in psychosynthesis contains:

The elementary psychological activities which direct the life of the body; the intelligent co-ordination of bodily functions. The fundamental drives and primitive urges. Many complexes, charged with intense emotion. Dreams and imaginations of an inferior kind. Lower, uncontrolled parapsychological processes. Various pathological manifestations such as phobia, obsessions, compulsive urges, and paranoid delusions. (Assagioli 2000, p. 15)

However, Assagioli’s model continues to further divide the personal unconscious into a hierarchy that includes not only the “basement of the house of the personality” but also “the attic with the window open to the sky” (Hardy 1987, p. 27). The middle unconscious (*2*) contains our awareness that lies within the periphery of our consciousness and remains “easily accessible to it” (Assagioli 2000, p. 15). This is where memories are held that are easily retrievable and where “imaginative activities are elaborated and developed in a sort of psychological gestation before their birth into the light of consciousness” (p. 15).

The higher unconscious or superconscious holds our greater human potential and is the region from which we receive our “higher intuitions and inspirations— artistic, philosophical or scientific, ethical ‘imperatives’ and urges to humanitarian and heroic action. In this realm are latent the higher psychic functions and spiritual energies” (Assagioli 2000, p. 15). Assagioli (2000) refers to the experiences of the superconscious as “transpersonal experiences,” which he states are universally described as being accompanied by the characteristics of joy, depth, ascent, expansion, empowerment, and awakening. Transpersonal experiences are how we occasionally glimpse and access our full creative, personal, and spiritual potential (Lombard and Müller 2016). Maslow (1968, 1971) described such experiences as “peak experiences,” which he associated with extreme inner health. He described them as including temporary disorientation with respect to time and space, feelings of wonder and awe, great happiness, and “the conviction that something extremely important and valuable had happened, so that the person is to some extent transformed and strengthened in daily life by such experiences” (1971, p. 164).

Viewed in this way, transpersonal experiences are far removed from Freud’s belief that they are merely the neurotic sublimation of the sexual urge or regression to the postnatal stage of development (Freud 1961). And although science and our rational selves might demand that we quantify and measure these experiences (e.g., MacDonald and Friedman 2002), Assagioli insists that the superconscious as a reflection of Self does not need to be demonstrated but is a fact of consciousness that contains its own evidence and proof within itself. Assagioli repeatedly stated that his principles and methods were based on solid personal experience (Haronian 1976), and he explained that transpersonal experiences are similar to experiencing:

a color, sound or feeling. It is neither possible nor necessary for anyone to ‘demonstrate’ the sensation of redness or greenness, joy or pain: for those who experience such things they are a psychological reality. (Assagioli 1993, p. 23)

This attitude is also reflected in Jung’s response at the age of 84 to the question, “Do you believe in God?” “I know,” he said, “I don’t need to believe. I know” (Burnett and Freeman 1959). Spirituality as a human experience and birthright is also eloquently expressed by Lewis (1940): “[Mankind] can close his spiritual eyes against the Numinous, if he is prepared to part company with half the great poets and prophets of his race, and with his own childhood, with the richness and depth of uninhibited experience” (p. 14). More recently, Freeman (2014) addressed the difficulties of including “the idea of transcendence with the province of psychology” and concluded that “listening to the claims of experience may pave the way toward a more inclusive, capacious, and adequate psychology” (p. 323). Finally, Haronian (1976) very clearly states, “Any model of human nature that attempts to be complete must include [transpersonal] experiences and offer an explanation for them” (p. 31).

### Psychosynthesis view of spirituality

As previously mentioned, one of the distinguishing concepts of psychosynthesis is that the Self and superconscious relate to the higher urges within human beings that allow them to develop their personality at different levels, including, but not necessarily, a spiritual one. In this way, spiritual drives and urges are as real and fundamental as, for example, sexual and combative drives (Assagioli 2000). In addition, psychosynthesis psychology views spirituality not necessarily as a specific religious experience (although it can be) but rather as a fully human and universal sense of being connected to a transcendent reality. Assagioli (2000) states that the word “spiritual” includes “all states of awareness, all the functions and activities which . . . [possess] values such as the ethical, the esthetic, the heroic, the humanitarian, and the altruistic” (p. 35). This definition agrees with that of Maslow (1954), who noted that mystical experiences, as described by James (1943), are a subset of the broader realm of peak experiences, which can also include nonreligious feelings of ecstasy, wonder, and awe.

### Personal psychosynthesis and spiritual psychosynthesis

As stated earlier, the aim of psychosynthesis is to establish the I–Self connection, and this process can develop along two lines: *personal psychosynthesis* and *transpersonal* or *spiritual psychosynthesis*. Human growth that involves work with either the middle unconscious or the lower unconscious is referred to as personal psychosynthesis. Therapeutically, the aim of personal psychosynthesis is to eliminate repressions, fears, and childhood dependencies; mature beyond self-centeredness; and learn how to change emotionally distorted outlooks into objective, sane, and rational considerations of life. The ultimate goal is that individuals be able to continually harmonize and integrate all contrasting, partly undeveloped, and uncoordinated conscious and unconscious functions to the point where they fully recognize their responsibilities in this world and appreciate others (Assagioli 2000). Spiritual psychosynthesis aims to integrate material from the higher unconscious into aspects of the personality (Firman and Gila 2002). Persons who undertake spiritual psychosynthesis begin to transform their behavior and attitude, becoming more compassionate and inclusive and less controlled by unconscious drives such as greed or rage (Assagioli 2000). Not for the meek-hearted, spiritual psychosynthesis is a dynamic and challenging process—“a long and arduous journey . . . full of surprises, difficulties, and even dangers [involving] a drastic transmutation of the ‘normal’ elements of the personality, an awakening of potentialities hitherto dormant” (Assagioli 2000, pp. 35–36).

Personal growth is related to spiritual growth in that, as individuals develop healthy, actualized personalities, they are then able to move beyond a sense of selfhood toward a broader and more universal understanding of their reality. Similar to Maslow’s hierarchy of needs, to which he ultimately added self-transcendence as the ultimate need (Koltko-Rivera 2006), personal psychosynthesis prepares the individual to enter into conscious communion with the deeper and more meaningful part of their lives, as expressed through such higher qualities as peace and altruism as well as the ability to engage more deeply in relationship. It is important to note that personal psychosynthesis alone can be satisfactory to many individuals and a worthy endeavor in itself. But for some, personal psychosynthesis is not enough. Others long for spiritual psychosynthesis, which allows them to relate to their higher urges and grow toward greater realization of their spiritual essence. Spiritual psychosynthesis does not skip over the work of personal psychosynthesis but attempts to accomplish both. Assagioli (2000) states that therapists who are spiritually inclined (such as pastoral psychologists) can be of great help to individuals who are “unconsciously groping” for this journey towards a higher reality (p. 50). These two developmental paths correspond with Maslow’s (1968) recognition that some individuals are “self-actualized” and others are “transcending self-actualizers,” the latter distinguished as people in touch with superconscious material or peak experiences.

Assagioli (n.d.)was fluent in Sanskrit and a scholar of Eastern religions, and he understood that the realization of the Self was a “supreme paradox” that could manifest through three distinct attitudes: (1) the Buddhist understanding of “no self”; (2) the mystical merging in another, in God; and (3) the Vedanta philosophical realization of the true Self, of one’s true being. He wrote:

If one identifies ‘Self’ with the empirical personality, then the attitude is either (1) or (2), according to whether one is mystical or not. If one identifies oneself with the emerging spiritual consciousness and transfers the self-identity to each higher level, then (3). There are advantages and drawbacks of each attitude. What is important is to recognize that the three attitudes are three ways of realizing the same glorious Reality, of attaining the same sublime goal. (n.d.)

Perhaps this inclusion of such distinct attitudes and their equal potential in helping us reach great inner freedom and joy is what makes psychosynthesis so fruitful. By including a higher consciousness within the psyche’s framework and a Self to which we all have a universal and yet personal connection, psychosynthesis concepts and techniques provide a framework in which the realization of the Self is not only permitted but nurtured, anticipated, and longed for.

Having described Assagioli’s model of the human psyche (as shown in Fig. 1), I now introduce two other central concepts that are essential to understanding psychosynthesis: the will and right relations.

### Will as energy

In psychosynthesis, the will does not correspond to the Victorian idea as something stern and oppressive. Instead the will has “a *directive* and *regulatory* function; it balances and . . . utilizes all the other activities and energies of the human being without repressing any of them” (Assagioli 2002, p. 10, emphasis in original). Assagioli describes the fully developed human will as having four aspects: the strong will, skillful will, good will, and transpersonal will (p. 15).

Most people associate the will with the single aspect of strong will. We need strong will to take necessary action, such as to do something we do not want to do or stop doing something that we want to do. But strength, when used alone without the other aspects, can actually cause harm to oneself and others. Skillful will “consists of the ability to obtain desired results with the least possible expenditure of energy” (Assagioli 2002, p. 15). Assagioli uses the metaphor of driving a car to explain the difference between strong and skillful will. With only strong will at your disposal, you would stand behind the car and try to move it by pushing with all your strength. Skillful will, on the other hand, involves knowing how to open the car’s door and sit inside, place the keys in the ignition, start the engine, and drive off. Assagioli (2002, p. 16) describes good will as “learning to choose right goals” (i.e., continuing with the car metaphor, to drive the vehicle in the “right” direction). As mentioned earlier, transpersonal will is the will of the transpersonal Self (i.e., driving the car as directed by the Self). Part of the work of a psychosynthesis counselor is to help clients to come into relationship with all four aspects of their will. For most of us, one aspect is more dominant, and the aim is for all four aspects to be equally developed and accessible within our field of consciousness and personal “I.”

Essential in the process of personal psychosynthesis is to understand that one’s will is pure energy that the “I” can direct conscious choices and actions in this world. In this way, the personal self and the will are intimately connected; that is, they are in relationship with one another. By gaining more awareness of how the will operates in one’s life, the individual can begin to exercise, strengthen, and call upon the aspects of the will that are most appropriate in any here-and-now situation. Spiritual psychosynthesis involves learning how to recognize, relate, and work with transpersonal will or, in other words, consciously working towards deepening the I–Self connection, which is itself another relationship between the personal self or “I” and the Self. Both the “I” and the Self, as previously described, are pure consciousness and will, whereas the “I” is a pale reflection of the Self. Pastoral psychologists can understand the Self as God’s consciousness and will and the “I” as the full human being that we are all called to become in baptism. The idea that the Self is both universal and individual can be equated to the Christian understanding that we are all created in the image of God; that is, we all reflect the universal higher qualities of God through our unique individual identities. Spiritual psychosynthesis is the journey towards strengthening the I–Self relationship or, in Christian terms, our relationship to God. Relationship is one of the keys to all psychosynthesis work and is further discussed in the next section.

#### Relationship model of the will of the “I” and the will of the Self

**Fig. 2:**
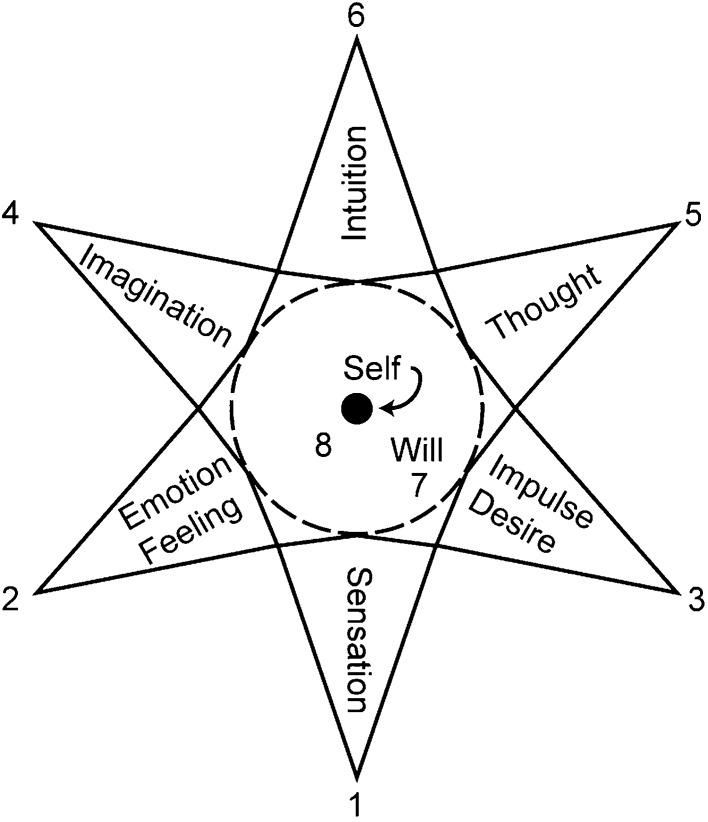
The relationship between the personal will and the transpersonal will and how they affect psychological functions (Assagioli 2002, p. 12)

Assagioli (2002) illustrated the relationship between the will of the “I” and the will of the Self, and how they affect the different psychological functions (see Fig. 2). He stated that the will (*7*) holds a central position of prominence. Through the will, the “I” regulates and directs the individual’s imagination, thoughts, emotions, etc. (*1–6*). Assagioli argued that once the “I” becomes more connected and in relationship with the Self (*8*), then the transpersonal will can also act on the individual’s various psychological functions and “transcend the limitations of ‘normal’ consciousness” as expressed through acts of love, action, beauty, and self-realization (2002, pp. 115–116).

#### Stages of an act of will

In addition, Assagioli (2002) states that any act of will has six sequential stages: purpose, deliberation, choice, affirmation, planning, and execution. Although not every stage holds the same importance in any single willed act, the “principal cause of failure in completing an act of will is that people often have difficulty carrying out one or another specific stage” (2002, p. 137). The role of the psychosynthesis counselor is, once again, to guide the client towards understanding which stage of their decision-making might be weaker than the others and to bring all these stages into relationship with the “I” and Self.

### Working in right relations

Throughout psychosynthesis, the essential dynamic of relationship permeates. The psychosynthesis counselor is continually working in relationship with the client, guiding the client to a fuller relationship to his or herself, others, and ultimately the Self. At the same time, the counselor is aware of her own relationship to herself, her client, whatever is manifesting in the counseling room, and the Self. This idea in psychosynthesis is called “right relations,” which refers to the use of all aspects of will and the deepest awareness possible to relate to all that is present in space and time. Right relations deeply depend on the synthesis of two guiding principles: love and will. Assagioli (2002) wrote

One of the principal causes of today’s disorders is the lack of love on the part of those who have will and the lack of will in those who are good and loving. This points unmistakably to the urgent need for the integration, the unification of love with will. (p. 91)

The first step is to develop the weaker of the two so that loving and willing become equally available. The next step is to then awaken and manifest the higher aspects of both. Finally, we must learn how to alternate between love and will in such a way that each arouses and reinforces the other. The task is to ultimately develop love and will in balance and strength. But even beyond that goal, one’s aim is not to compromise either love or will but instead to synthesize them into a higher unity that transcends the qualities of either. In other words, to be compassionate is not enough; we need acts of compassion, a definite synthesis of love and will. Psychosynthesis views working in right relations as such an act. The union of love and will fosters and acts upon the growth and evolution of individuals who are in relationship.

Relationship as a manifestation of love and will is one of the most essential qualities of psychosynthesis counseling work. The psychosynthesis orientation in the counselor-client relationship is an attempt to create a real, open, and honest space for love and will to synthesize and allow for personal and spiritual growth. Through such a therapeutic relationship, the client can learn to relate to his or her inner and outer worlds (and to God) in a new way. The goal of developing personally and spiritually within such a therapeutic alliance can be challenging for both client and counselor, as they inevitably enter into the realities of transference and countertransference (Jung 1966; Spiegelman and Mansfield 1996). However, through such a therapeutic alliance, new and more satisfying ways of being in relationship can emerge for the client and, in turn, impact his or her other relationships. According to psychosynthesis, each of us has the responsibility to become aware of our situation and choices, act with good will, and work towards positive growth—individually, socially, and universally. Ultimately, one of the long-range goals of psychosynthesis is to establish a full, permanent sense of relationship with all beings (Haronian 1976; Roex 1988).

In fact, it is through an empathic connection to others that we receive an awareness of ourselves as whole—that is, the awareness of the authentic personality or “I” (Firman and Gila 2002). The goal of all psychosynthesis counselors is to become an empathic mirror for clients in order to facilitate the blossoming of their authentic personality. In psychosynthesis, those people who empathically attune to others are able to manifest the I–Self connection as an external unifying center. Ultimately, the goal in therapy is for the client to internalize that unifying center by rebuilding their own I–Self connection as they heal earlier wounds and redeem their authentic personality. All this healing and growth can only happen in right relationship. This concept of right relations corresponds precisely with the ultimate goal of any pastoral psychologist—to be able to become sensitive to others, comprehend and experience difference, maintain an authentic self-identity, and form a relationship with God.

### Psychosynthesis techniques

Psychosynthesis counseling techniques include guided visualization, daily self-reflection, role-playing, ideal models, symbolic images, storytelling, free-drawing, and dream work. Although psychosynthesis counseling sessions might look similar to other forms of counseling, such as psychodynamic, person-centered, or Gestalt, the distinct aspect of psychosynthesis is the idea that there is a Self and that all counseling is ultimately trying to achieve the recovery of the Self (Whitmore 2004). In addition, psychosynthesis counseling explores issues beyond the behavior patterns and symptoms of the client’s presenting issue. This approach includes attempting at all times to work alongside the Self by creatively holding the space in which the client’s problems can be aired and consequently the spiritual context, that is the voice of the Self, can emerge (J. Evans 2007; R. Evans 2007). The counselor’s task is to assess the client’s presenting issue in the here-and-now to understand what the Self is calling the individual’s personal “I” to heal and redeem. Working within such a context is described as “bifocal vision,” which is to see “the difficulties and suffering experienced in the personality [i.e., the client’s symptoms] . . . as the urge to wholeness, to expression, and realization of Self” (J. Evans 2007, pp. 75–77). Throughout the process of psychosynthesis, the will is continually evoked and strengthened. Strong will is often required to examine one’s inner nature in a sincere way. Skillful will is needed to disidentify from those feelings, thoughts, and roles that dominate us, and good will is vital when selecting the most appropriate behavior in the present context.

Ultimately, psychosynthesis counselors work within a flexible yet structured framework that helps clients to examine and ruminate over the situations that trigger their problems, the interpersonal relationships involved, the physical sensations and emotions evoked, the attitudes and beliefs stimulated, and the values that may be hidden and implicit (Assagioli 2000; Nguyen 2002; Whitmore 2004). Problems and obstacles are seen not as pathological states to be eliminated but rather as creative opportunities that “at their deepest level are inherently meaningful, evolutionary, coherent, and potentially transformative” (Whitmore 2004, p. 11)**.**

#### The self-identification exercise

The self-identification exercise, also referred to as the disidentification exercise or the body-feeling-mind meditation, is a fundamental psychosynthesis technique for working with all aspects of the will. The goal of the self-identification exercise is to systematically connect and bring awareness and affirmation to the physical, emotional, and mental aspects of the personality and then guide the client to disidentify from each aspect and connect to the “I,” the source of pure consciousness and will. The guiding psychological principle of this exercise is: “We are dominated by everything with which our self becomes identified. We can dominate and control everything from which we dis-identify ourselves” (Assagioli 2000, p. 98).

For example, after acknowledging that we *have* feelings, disidentification occurs when we further understand that we *are not* our feelings, but, in fact, are much more than this single component of ourselves. Even though we may derive our greatest sense of personal identity from, for example, our body (or parts of our body), specific feelings, and/or transitory thoughts and attitudes or life roles, that viewpoint is in fact limited in scope and impossible to maintain, given that life is transitory in nature. Our physical sensations, emotions, and thoughts are continually renewing and changing while we remain at our core the same “I.” By learning to disidentify from these aspects of body, feelings, and mind, we can then begin to dominate, discipline, and deliberately use them by way of our authentic personality—the simple, unchanging, and self-conscious “I.”

#### The subpersonality model

Psychosynthesis assumes that we all have multiple subpersonalities, such as Mother, Daughter, Teacher, and Leader, that help us to function in the world, mostly without much reflection or conscious choice. Often these subpersonalities are polar in nature, acting contrarily with antagonistic traits. We might be carefree and spontaneous in one situation and frozen in another. Perhaps most essential is the notion that a higher quality lies at the core of each subpersonality, no matter what its outer behavior might be. These higher qualities, like truth, strength, and courage, are considered to be transpersonal, universal, and timeless. However, these qualities can often be degraded or distorted when expressed through a subpersonality. The challenge is not to repress or eliminate any subpersonality’s behavior but rather to recover its higher quality and express that gift in a more positive and holistic way. Psychosynthesis is about synthesizing all one’s subpersonalities into a unifying center of authenticity where the “I” becomes the director and observer of all subpersonalities, enabling them to function in a harmonious and balanced way. The subpersonality model includes the following stages: recognition, acceptance, coordination, integration, and finally synthesis of one’s numerous subpersonalities.

By working through the subpersonality model process, the roles that we play in our lives can become synthesized into a unifying center of authenticity. As integration of an individual’s subpersonalities proceeds and personal psychosynthesis develops, less energy is lost in managing conflicting subpersonalities—instead, their potential strengths and capabilities become available. With subpersonality integration, one’s personal field of consciousness expands to include those subpersonalities that were previously acting unconsciously and, consequently, the individual gains more access to creative material in his or her middle and lower unconscious. Ultimately, synthesis of one’s subpersonalities allows for the greatest freedom of expression, as creative intuitions that exist in the unconscious can be more readily actualized by the directing “I.”

#### Spiritual psychosynthesis techniques

Psychosynthesis counseling techniques for helping clients to achieve personal psychosynthesis, such as the self-identification exercise, are essentially the same as those for achieving spiritual psychosynthesis since interventions that enable an individual to develop on a personal level also include those methodologies that enable the “I” and superconscious to interact. For example, the self-identification exercise is also used to disidentify from transpersonal experiences and strengthen the personal “I” so that the individual might not become fixated by or identified with such experiences. Assagioli (1993, 2000) has defined techniques for helping one achieve spiritual psychosynthesis. These include the use of specific symbols (for example, the sun or the blossoming of a rose); inner dialogues (for example, with the Inner Christ); reading specific texts, such as Dante’s *Divine Comedy*; and listening to music, prayer, and meditation. In addition, in his seminal essay “Self-Realization and Psychological Disturbances,” Assagioli (2000) clearly defines four critical stages of spiritual awakening: (1) the crises preceding the spiritual awakening, (2) the crises caused by the spiritual awakening, (3) reactions following the spiritual awakening, and (4) phases of transmutation. He ends his essay with considerations for those who guide persons undergoing a spiritual awakening, including the understanding that the physical, emotional, and mental disturbances that arise after such incidences are temporary reactions of inner growth and regeneration.

For a full explanation of psychosynthesis counseling techniques, please refer to the literature (Assagioli 1993, 2000; Carter-Harr 1975; Crampton 1970; Ferrucci 1982; Firman and Gila 2002; Firman and Vargiu 1974; Haronian 1976; Lombard 2014; Lombard and Müller 2016; Vargiu 1974; Whitmore 2004).

## Psychosynthesis counseling examples of spiritual and personal growth

In previous studies, psychosynthesis techniques were shown to intervene, relieve, and transform the effects of culture shock experienced by student sojourners in the Netherlands (Lombard 2014) and to open the door to their creativity (Lombard and Müller 2016).

This article further describes how psychosynthesis counseling aided some of these clients to become aware of their spirituality and to practice techniques that allowed for superconscious material to emerge.

### Research method

The source of the data is psychosynthesis counseling work conducted in the Netherlands from October 2008 to November 2013. The data gathered and analyzed were qualitative, and the following is a brief outline of the research method employed. For a full explanation of the participants, psychosynthesis clinical techniques, data collection, and data analysis, please refer to studies by Lombard (2014) and Lombard and Müller (2016).

#### Participants

A total of eleven clients (two male and nine female) voluntarily sought counseling, ranging in age from 25 to 36 years old. All were international scientists working in technical fields (see Table 1).

**Table 1:** Participant details

Pseudonym	Nationality	Gender	Age at the start of therapy	Research area	Religious identification	Number of sessions
Liliana	Italian	Female	28	Philosophy of technology	Atheist	55
Nicole	French	Female	26	Philosophy of technology	Atheist	42
Maria	Portuguese	Female	26	Tissue engineering	Atheist	41
Paulo	Brazilian	Male	29	Computer science	Atheist	34
Henk	Dutch	Male	27	Tissue engineering	Atheist	32
Susan	Canadian	Female	28	Philosophy of technology	Atheist	31
Julia	Austrian	Female	29	Communication science	Atheist	25
Biyu	Chinese	Female	29	Nanobiophysics	Atheist	13
Jingfei	Chinese	Female	30	Spatio-temporal analytics, maps, and processing	Uncertain	12
Thabisa	South African	Female	36	Sustainable production and consumption of energy	Christian, prays occasionally	10
Carol	South African	Female	31	Biophysical engineering	Prays but doesn’t know to whom or what	10

#### Psychosynthesis clinical methodology

Clients met the counselor from 10 to 55 times, two to four times per month, with each session lasting one hour. In total 305 h of counseling work occurred. Sessions were conducted in English. In addition, clients were invited to self-reflect and write about any critical issues they faced or emotions they felt between sessions and to email their observations to the counselor. Although the subject for self-reflection remained open at all times, the client would sometimes be directed to reflect and write on a specific topic and email that reflection before the next session. All clients were assured of confidentiality and anonymity, and permission from all clients to quote them in this article was obtained.

All the psychosynthesis techniques presented in the introduction were employed during the counseling sessions as the counselor deemed appropriate, but the dominant techniques used were the self-identification exercise and the subpersonality model. During the initial sessions, the clients were led through the self-identification exercise. Unlike other meditation techniques such as mindfulness, the self-identification exercise is grounded in the theory of psychosynthesis and has the specific purpose of helping the individual become more of an observer and director of all the personality’s aspects and activities. The self-identification exercise is easily executed by clients on their own, and to help them to do so, all the clients were provided with a recording of the meditation. During subsequent sessions, the counselor would encourage daily practice of the exercise by asking about the clients’ progress and discussing possible solutions to any obstacles. For a full text of this meditation and a full explanation, please refer to Assagioli’s description (2002, pp. 211–217) and Lombard (2014).

With regard to the subpersonality model, during psychosynthesis counseling sessions, clients initially recognized their subpersonalities by assessing, alongside the counselor, which subpersonalities might be playing a dominant role in their presenting issue(s). These subpersonalities were revealed through the different roles clients played in different situations with different people. Once the subpersonality was recognized, the next step was to give it a name—for example, the Child or the Artist. Humor was used during this stage to facilitate disidentification, allowing the client to playfully engage in relationship with the subpersonality. After naming the subpersonality, the client then created its character sketch, including a drawing of the subpersonality, and was asked to continue reflecting upon it in a personal journal.

After recognition, the next step for the client was to accept his or her subpersonality. The client was asked to observe what triggered each subpersonality’s appearance and to watch and allow that subpersonality to exist. This exercise helped to strengthen the observer “I.” Alongside acceptance was the complementary stage of coordination, which had the basic purpose of identifying and becoming more conscious of the subpersonality’s wants and needs in order to find more acceptable ways in which the needs could be fulfilled. Therefore, clients first identified how the needs of a subpersonality were typically fulfilled. Subsequently, clients were asked to imagine how they might fulfill that subpersonality’s needs in new, objective, and creative ways in order to transform inner conflicts.

The final stages of the subpersonality process—integration and synthesis—are lifetime endeavors. Although coordination deals with the development and understanding of specific subpersonalities, integration is concerned with the relationship between subpersonalities as well as each one’s activity within the personality as a whole. Synthesis involves the culmination of individual growth that allows for balance and harmony of the entire personality and is essentially interpersonal and transpersonal. As a result of synthesis, the life of the individual and his or her interactions with others become “increasingly characterized by a sense of responsibility, caring, co-operation, altruistic love and transpersonal objectives” (Vargiu 1974, p. 89).

To initiate these final stages, clients were presented with an opportunity for their subpersonalities to interact. Numerous techniques were used to allow for such interactions, including guided visualization, role-play, imaginary meetings, and/or letter writing from the observer to the subpersonality (and vice versa). Throughout such interactions, clients were encouraged to strengthen their role as the observer and, consequently, to consciously and more creatively fulfill any conflicting needs. Clients were ultimately guided to assess, appreciate, and come into relationship with the higher quality held by each of their subpersonalities. Once the need of the subpersonality was met in a new, creative way, clients were asked to reflect upon, practice, and observe their expression of the subpersonality’s higher quality in the world.

#### Data collection and analysis

Data collected and analyzed from the counseling sessions included emailed reflections, drawings created by the clients during the sessions, verbal testimonies of the six participants who were recorded, and the researcher’s reflections, which were carefully written and compiled immediately after all nonrecorded sessions. To initially assess the clients’ religious identification, past experiences with superconscious material (i.e., peak experiences), and awareness of a Self, the counselor asked the following questions during the initial interview: “Do you have any religious or spiritual practice?” and “Have you ever experienced a time when you felt like you were a part of or connected to something greater than yourself?” During the counseling sessions, however, the counselor did not ask any further questions about religion or religious practices, nor did she evangelize in any religious way or educate clients about the Self or superconscious. Instead, she applied the various psychosynthesis techniques to the client’s presenting issues and continually focused on holding the spiritual space that might allow the Self to emerge and the client to innately discover his or her spiritual dimension.

Work on the data was an evolutionary process that included written observations, reflections, and detailed notes of all meetings immediately after each session. Whenever a client revealed a new awareness of his or her spirituality, the counselor carefully noted the client’s emerging spirituality by logging the date, session number, and the client’s testimony verbatim. In addition, the clients themselves also played a central role in the process of data analysis in order to check for counterindications of the emerging theses (Yin 2003). Whenever a client spoke about his or her spirituality, the counselor further discussed the client’s observations in order to reach a consensual understanding. In this way, the clients were able to correct, reshape, or contextualize the counselor’s perceptions as they deemed appropriate, and their reflections and adjustments were integrated into the final data collection and analysis. The researcher listened to and reviewed the tapes of those clients whose sessions were recorded. Consequently, the narrative of each client was carefully held, reflected upon, observed, and analyzed with the aim of understanding the emergence and evolution of spirituality in each client’s psychological process.

#### Research ethics

It is important to note that the researcher and the counselor (as well as the author) are the same person in this study. This combined role of being both counselor and researcher can foster some inherent dilemmas, such as in cases where the client might want to please the therapist with their responses or the possible conflict between the therapist’s loyalty to her client vs. her research. Nevertheless, studies have shown that it is possible to combine the roles, and, in fact, a number of clinical researchers are of the opinion that such research in itself has a beneficial therapeutic effect on the patient (e.g., Lorentzen 2003; Richardson and Reid 2006; Sandahl and Wilberg 2006). Sandahl and Wilberg (2006) state that “it is not only possible, but also very necessary to unite the roles of researcher and clinician if we wish to develop applicable knowledge on psychotherapy” (p. 409). Many studies have appeared in the literature in which the researcher, counselor, and author are one and the same person (e.g., Jernigan 2001; Koppel 2004; Selvam 2015; Turp 2002).

To minimize any conflict, at the end of their last session, all clients were given the option to withdraw their testimony from this study’s final research results. In addition, at least six months after their therapy had ended, clients were sent an email asking them to answer the following five questions: (1) Did you know that the counselor was a psychosynthesis counselor? (2) Did you know that psychosynthesis was a spiritual approach? (3) Did the counselor ever bring up spirituality or did these types of questions come from you? (4) Did the counselor ever impose her values on you in any way? and (5) Did you ever feel that the counselor was “leading you” towards spirituality, religion, Christianity, or God? Clients were invited to either answer “yes” or “no” to each question and/or write freely in response to any of the specific questions or in general. One month after the first email was sent, a second request was sent by email to those clients who had not already responded.

### Results

The following sections describe the results found in this study. First, clients’ testimonies regarding their religious identities and past experiences with superconscious material are presented. Secondly, during the course of their counseling, three clients talked about their newly discovered spirituality and the actions they were taking with regard to this awakening, and their testimonies are presented. Then, testimonies from other clients about their personal growth are included, as these findings show an expression of qualities derived from the higher unconscious, such as strength, peace, and acceptance, as well as the ability to engage more deeply in relationships. Finally, the results from the email questionnaire that was sent to the clients once their therapy had terminated are presented.

#### Clients’ prior experience of superconscious material

When asked during the initial interview “Do you have any religious or spiritual practice?,” eight of the eleven clients said that they had no religious affiliation or belief in God and described themselves as atheist (see Table 1). Thabisa said that she did pray as a Christian on occasion but did not have any regular spiritual practice. Carol said that she longed to be spiritual and often prayed, but she did not know “to whom or what.” Jingfei, who was suffering with uncompensated labyrinthitis (better known as vertigo), admitted to having conflicting feelings regarding faith:

My grandmother and mother tried to make me a Buddhist. But I understand that if you are a Buddhist, then you only have your own inner being to depend on. With Christianity, there is always a God that you can go to. When I experience vertigo, I often imagine God right above me.

Of the eight others, the following testimony was a typical response:

I would call myself an atheist. As a scientist, I know that there is no proof showing that God exists. But I also know that there is no proof showing that He does not exist. (Henk)

Interestingly, clients’ responses became very different when asked if they had ever had a feeling of connection to something greater than themselves. Without exception, all had had a transpersonal or peak experience at some point in their lives, mostly while they were in a natural setting. In fact, James (1943) noted that nature “seemed to have a peculiar power of awakening such mystical moods. Most of the striking cases which I have collected occurred out of doors” (p. 394). Henk, who is quoted above, said:

When I was 20, I was on a boat at night and all around in the water were bioluminescent plankton. It was so beautiful; I became very emotional and cried. I wish my girlfriend had been there so I could have shared such a deeply moving experience with someone.

Upon being questioned, Maria’s face completely changed, from being tense and drawn to radiant and smiling, when she related this experience while in nature:

I had a deep connection while swimming in a lake in Finland under the stars. There were many experiences like this while I was recently traveling in Norway. I don’t believe in God, but I admit there are times when I think there might be a superior cosmic intelligence capable of creating this natural beauty.

Biyu related her experience of this type of connection when she was a child playing and sharing time with her mother. Raised Roman Catholic, Paulo insisted that he was an atheist, something which he emphasized a number of times during his meetings over the course of two years. However, he responded to the second question by relating a strong mystical experience he had had at the age of ten:

I was in the church at school during prayer time. I was holding my hands out and felt energy and light stream into them.

Similarly, despite having peak experiences as a child, Liliana talked about her choice to stop believing in God, after which time she no longer experienced superconscious material:

When I was younger, around 11 or 12, I had many [peak experiences], but then I took a pragmatic decision. I had to decide: I believe in God or I don’t believe in God. And I have to be consistent with that decision. I was beginning to become interested in philosophy and, of course, my own sexuality was starting to emerge. So I choose not to believe.

#### Clients’ spiritual growth

After a number of sessions, three of the eleven clients showed evidence of a more active spiritual life. All three had initially described themselves as atheists. The following testimony occurred during Maria’s 24th session:

I don’t know how to say this, but I’ve become more spiritual. More in touch with me. I now have a small space on the balcony of my house that I call my temple with my painting stuff. It’s really perfect. I already see many changes inside me. I’m much more in peace in my daily life. . . . I am much more connected to myself.

**Fig. 3:**
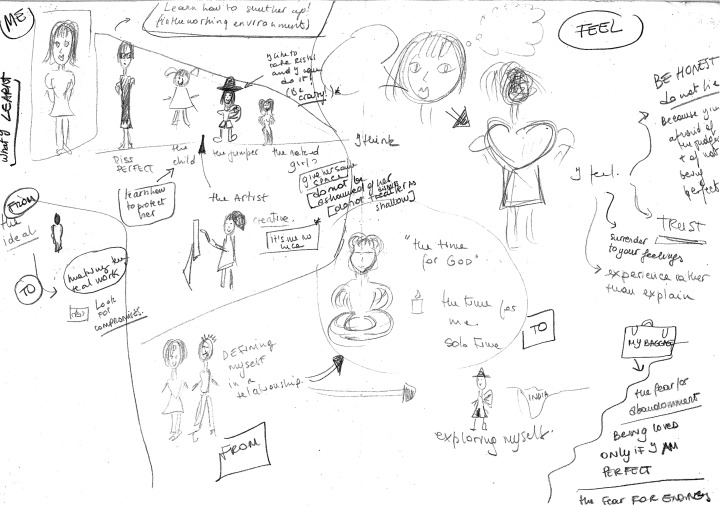
Client’s drawing of inner psyche with subpersonalities and “God Time” in the center

The second testimony comes from Liliana, who related her choice as a preteen to not believe in God. Before her final session, at the request of the counselor, Liliana made a drawing of her inner life (see Fig. 3). In this drawing, Liliana depicted the subpersonalities she had learned to coordinate and integrate (i.e., Miss Perfect, the Child, the Jumper, the Artist). Remarkably, at the center of the drawing she also depicted herself in a meditative state next to a lit candle. Next to this figure, she wrote “the time for God,” and directly underneath, “the time for me.” When asked about this figure, she said:

That’s my God time. It’s the time I make for God. Not in a religious sense, but in a spiritual one. It’s when I try to be quiet and not do anything. Just connect to God. Just connect to me. To myself. And I do this more now. It’s important. It gives me a sense of peace.

The final example comes from the client Julia, who, for the first time in her life, made plans to attend a two-week retreat at a monastery in order to actively pursue her personal and spiritual growth.

#### Clients’ personal growth

Although not all the clients spoke about their newly activated spiritual lives, the following testimonies clearly indicate that personal psychosynthesis occurred:

The ongoing therapy was very helpful to my personal growth. I’ll continue to cultivate the strength, calmness, peace, passion, and love I have learned with care and let them stay and grow deep inside me to illuminate my life and loved ones. (Biyu)

Our sessions helped me to grow personally far beyond what I could have predicted. (Maria)

What I have done . . . is a journey into self-exploration. It has been a process of understanding, acceptance, and management of different aspects of myself. This is a lifelong journey that I think everyone should begin. The counseling helped me immensely, not only in dealing with such a process but also in growing as a person and as a researcher. (Susan)

I’ve changed so much with your help. I’m now not just a better researcher but also a better friend, son, and brother. And the change is deep inside me. I know in the future that I will also make a better husband and father. You gave me the tools and techniques to make real choices. And no one can ever take these things away from me. (Paulo)

#### Questionnaire results

Eight of the eleven clients responded to the questionnaire after the second email was sent. Of the eight respondents, two clients did not know the counselor was a psychosynthesis counselor and one only knew this after the first session. Five clients did not know that psychosynthesis was a spiritual approach. Responses varied to the question “Did the counselor even bring up spirituality or did these types of questions come from you?” Four said “no,” one could not recall whether she or the counselor had raised the issue of spirituality, and three said “yes.” The three who said “yes “ all recalled spirituality being raised by the counselor only during the first session. Examples include the following feedback from Julia and Liliana, who were two of the three clients who demonstrated spiritual growth:

We discussed my take on religion and God once, but I feel this was brought up by [the counselor] just to get a better sense of my personal history, but I didn’t feel it impacted my relationship with her during our process or the result of my work with her. (Julia)

During our very first session, the counselor asked me about my relationship to God. I told her I didn’t have a relationship with God and I didn’t want to have one. I felt that she accepted it, and we didn’t talk about religion and God as such anymore during our sessions. (Liliana)

All eight clients responded “no” to the two remaining questions: “Did the counselor ever impose her values on you in any way?” and “Did you ever feel that the counselor was ‘leading you’ towards spirituality, religion, Christianity, or God?” Three clients clearly stated they would not have continued with their sessions if they had felt “led” by the counselor towards spirituality:

If I felt that she was imposing her values (and not those of her profession), I would have stopped attending sessions with her. (Thabisa)

I didn’t feel the intentional leading from my counselor towards religion, Christianity, or God (and I would not have allowed it). (Biyu)

I am an atheist and I really appreciated that religion or God were not “part” of the sessions. Maybe [the counselor] sensed that (I am not into religion and spirituality, to put it bluntly); and I am very grateful for her sensitivity and for the fact that, even during moments when spirituality or God could have been mentioned (such as during meditation or for self-understanding), I never felt that I was being invited or pushed towards spirituality, religion, Christianity, or God. (Nicole)

## Discussion

This article attempts to offer psychosynthesis concepts and its model of the human psyche as a foundational bridge between psychological and spiritual conceptions of human nature. By recognizing the existence of a higher consciousness within all human beings, psychosynthesis is an open system that defines the human being as a psychological and spiritual being. Nondogmatic and universal in its approach and conceptual understanding of human nature, the orientation of psychosynthesis therapy is based on our having an inherent need to grow, personally and spiritually, and supports this positive evolutionary process (Haronian 1976). Results in this study included testimonies from clients who expressed not only their experience of personal growth but, in some cases, newly gained spiritual awareness and their longing to continue to develop this dimension of their lives. In addition, the eight clients who responded to an email questionnaire (including the three clients who exhibited spiritual growth) did not feel that the counselor ever imposed her values or “led them” towards spirituality, religion, Christianity, or God. Perhaps of particular interest to the pastoral psychologist is that the three clients who felt an inner need to further explore and develop their spirituality identified themselves as atheists. This activation of spirituality in self-identified atheists can lead to the assumption that, for those psychologists working with faith-based clients, psychosynthesis can provide a powerful platform from which to guide them towards spiritual growth.

### Psychosynthesis as a model for spiritual psychology

According to Helminiak (2009), we are in need of an explanatory science of spirituality that explores the structures, mechanisms, or processes of spiritual integration and growth. He actually calls for a type of equation to encapsulate spirituality the same way a Pythagorean theorem provides an equation for a perfect right triangle. The Pythagorean theorem is an abstraction to help our understanding of right triangles even though it “looks nothing like a triangle in the physical world” (Helminiak 2009, p. 164). I propose that Assagioli’s model of the human psyche be considered as a possible formula for spiritual psychology. Fundamental to his model is the I–Self connection—the ultimate alignment of the will and consciousness of the personal self with the will and consciousness of the Self, the ideal reality towards which we all strive as human beings. One could interpret Liliana’s placement of the text “the time for God” directly above and not too distant from (i.e., in relationship to) “the time for me” in her drawing (see Fig. 3) as a reflection of her inner longing for the I–Self connection depicted in Assagioli’s model.

For Christians, this perfect I–Self connection is manifested in Jesus, who is the highest and fullest embodiment of what is both essentially human and divine, and as such, Jesus represents the perfect model of personal and spiritual psychosynthesis. Also fundamental to Christian faith, the idea of relationship is paramount to the process and outcome of full personal and spiritual psychosynthesis. The Christian faith considers God to be a triune God, a Trinity of divine Persons whose interrelationships are mirrored by the relationships involved in the triple (not double) commandment of love: “Love the Lord your God with all your heart and with all your soul and with all your mind and all your strength. And … love your neighbor as yourself” (Mark 12:30–32, NIV). Christians are called to come into relationship with God, their fellow human beings, and themselves through their relationship with Jesus: “As the Father has loved me, so I have loved you. Now remain in my love. If you keep my commands, you will remain in my love, just as I have kept my Father’s commands and remain in his love. . . . This is my command: Love one another.” (John 15:9–10, 17). Jesus’ command to to willfully love and have a loving will corresponds to the four aspects of the will as defined by psychosynthesis and the further relationship of our free will with God’s will as the essential activating forces upon our psychological functions (see Fig. 2).

Helminiak (2009), however, warns us that “any psychological construct that includes God as an essential element inevitably results in a muddle” (p. 165). This muddle derives from the confusion created by ambiguous terminology such as spirituality, God, self-transcendence, and relationship to God; how to assess and measure such terms; and where to place such constructs in the science of psychology. Once again, psychosynthesis attempts to “unmuddle” these concepts by stepping beyond the idea that the Creator (Self) and the created (“I”) are mutually exclusive. Through its definition of the Self as always in relationship with the “I,” psychosynthesis requires us to move outside of a one-dimensional, digital mode of thinking into a multidimensional and relational mode of thought that allows for an interdependent and analogical relationship between all things. Assagioli (1980) explains that although there appears to be a dualistic separation between our ordinary personality and the Self, as if the Self were external and superior, “the reality is that WE ARE the Self; that is, the part of us that is most true, most real, is the spiritual Self” (p. 32, emphasis in original). Assagioli asserts that his model clearly shows this paradox with the positioning of the Self both outside and inside the interior psyche. He further explains:

It may appear to be a contradiction . . . but the Self is both individual and universal. It is individual in that it is the part that is the most real and intimate, the most “individually unique” within us. But it is universal in that it is not limited within or by our transient personality, nor does it involve the continuous influx of our psyche or conflicts, or the combination of various energies and activities of our body, feelings, or mind. (p. 32)

Here, one may think of what is perhaps the most famous of Augustine’s sayings: “I was seeking you, God, but you were deeper in me than my inner being itself and higher than my very heights” (*Confessions* III.6.11).

The testimonies of Liliana and Maria, who both discovered a spiritual dimension in their lives after receiving psychosynthesis counseling, exemplify the existence of the Self as both universal and individual as well as its relationship to the “I.” One could transcribe both Liliana’s and Maria’s testimonies slightly differently by replacing “myself” with “my Self.” For example, Liliana said: “That’s my God Time. It’s the time I make for God. . . . Just connect to God. Just connect to me. To myself.” And Maria: “I am much more connected to myself.” Similarly, Jingfei spoke of imagining God right above her, especially when she felt in need of help during episodes of vertigo. Interestingly, she also articulated one of the essential aspects of psychosynthesis that best applies to pastoral psychology: “With Christianity, there is always a God that you can go to.” Within the psychosynthesis context, the I–Self connection can be viewed as one’s relationship with God, and this connection is always available. As stated in the introduction, the “I” is a direct reflection of the Self. Although the “I” is the essential consciousness and will of the human individual, it remains directly and “indissolubly united to Self as a mirror image is indissolubly united to that which it reflects” (Firman and Gila 2002, p. 39). Assagioli (1993) further explains that this profound relationship is constant; the Self is “in a continuous relationship with the individual’s superconscious—and partly outside of that [individual’s] personality” (p. 31). This understanding expresses the dual nature of the Self. Like God born as Jesus in human form, the Self is both universal and individual at one and the same time. As human beings, we too share His image (also as a pale reflection!) and spiritual heritage.

Finally, the Self as both universal and individual appears in this study’s results in two ways. Graphically, consider once again Liliana’s figure of herself in a meditative state. This figure, of course, is a sketch representing her inner life, but it is interesting to note how universal this figure appears when compared to the other figures in her drawing. Outside the context of the drawing, this central figure could be male or female, old or young, Western or Eastern. This universal yet individual nature of the Self is also represented in the client sample presented in this article, which included nine nationalities across four continents and encompassed a wide range of cultural identities, values, and belief systems. Despite these vast differences, the clients’ experiences of psychosynthesis were similar; all grew in personal consciousness and will (the “I”) and, consequently, were more able to observe, identify, and direct their own wants, needs, emotions, intuitions, and imagination. In addition, all were better able to more freely and consciously choose and execute their will and to interact with others in constructive and positive relations. The three clients who experienced spiritual psychosynthesis were also similar in their pursuit of activities such as meditation, prayer, and a search for meaning in their lives.

### Implications for the Christian psychosynthesis counselor

As the psychosynthesis counselor, I found that my most important task was, in fact, to hold the space during each therapy session so that the Self might be able to emerge within the context of the client’s issue in the here-and-now. By “hold the space,” I mean that I attempted to consciously remove any obstacles such as assumptions, judgment, or fear within myself as the therapist and within the therapeutic relationship. Through activation of a loving will, I hoped to be able to recognize the will of the Self in terms of the personal and spiritual opportunity for growth (that is, further alignment of the I–Self connection) within the client’s immediate suffering. Such an open attitude allows for imaginative insights, intuitive understandings, and the emergence of emotions and thoughts that might have been previously repressed or unrecognized, as well as bodily sensations that can unveil vital information about the prevailing issue to both the therapist and the client. In other words, “holding the space” is a conscious and willful act in the attempt to work together with the will of the Self through the psychological functions of both client and counselor (as shown in Fig. 2). As a Christian, I personally imagined and experienced this space left open for the Holy Spirit to enter and lead us forward in healing, authenticity, and love. The fact that none of the eight clients who responded to the questionnaire felt that I, as the counselor, had imposed my own personal values nor “led” them towards spirituality is strong indication that the role of the counselor is perhaps best served through “holding the space” by which the Holy Spirit can emerge and, in its own mysterious way, bless the therapeutic relationship and allow both the counselor and client to personally and spiritually grow.

### Limitations and future research

This article has some limitations that need to be addressed. First and foremost, it does not provide a hermeneutic discussion of a possible integration of psychology and spirituality within the psychosynthesis context nor of an integration of psychosynthesis and the Christian faith. In this article, I have limited myself to suggesting the convergence of the latter two perspectives; future studies should address this issue extensively. Nevertheless, not only does psychosynthesis resolve the tensions between Freudian psychoanalysis and religion, but its adherents specifically contend that spiritual drives are as real and fundamental as sexual and combative drives. Open to the spiritual dimension in this way, psychosynthesis concepts further connect to Christian faith and theology through a rich tapestry of ideas discussed in this article, such as the I–Self relationship as manifested in Jesus and the way in which His full embodiment of what is essentially both human and divine can be a model of complete personal and spiritual psychosynthesis; the four aspects of will, including the transpersonal or God’s will; the synthesis of love and will; and the paramount importance of being in relationship to all living things through a triune God.

These concepts were further supported by the qualitative data of the testimonies of clients who received psychosynthesis counseling. However, the small client sample, although culturally and demographically diverse, makes it difficult to draw any general conclusions. Future research in this area should try to increase the number of participants. The number of sessions for each client also varied widely (10–55), which might have influenced the psychosynthesis process. However, the data does demonstrate that psychosynthesis counseling positively influenced the personal growth of all clients and the spiritual growth of three of the clients. Noteworthy is the fact that the three clients who experienced spiritual growth did not reveal this new dimension of their lives until they had had at least 24 sessions with the counselor, indicating that spiritual growth needs time to develop and/or to share with another.

Another limitation is the qualitative research method used in this study. There is a long debate in the literature on whether or not qualitative research can or cannot indicate causal relationship (see Maxwell 2004a). Nevertheless, in line with the realist position (Maxwell 2004b) (especially when it comes to the process of giving meaning to certain events as psychosynthesis does), cause-effect relationships can be drawn. Future research should, however, investigate the effectiveness of psychosynthesis counseling in spiritual growth by collecting empirical data and using well-established tests that measure spiritual awareness, religious participation, and commitment to a religious belief system, such as the Revised Religious Life Inventory (RLI-R; Hill et al. 2005). The qualitative methodology of this study, however, did allow for intimate conversations with clients over many months and brought depth and breadth to the analysis that quantitative analyses alone cannot provide. In addition, the qualitative approach in this study was especially conducive for conducting research into spiritual identity and personal and spiritual psychosynthesis, as this approach matches the methodology of interpretative phenomenological analysis (IPA). IPA relies on small sample sizes (5–10 participants) as it “is committed to understanding how particular experiential phenomena (an event, process or relationship) have been understood from the perspective of particular people, in a particular context” (Smith et al. 2009, p. 29). Finally, the qualitative approach to this study is perhaps more aligned with the approach taken by pastoral psychologists.

Future studies might include investigations into how psychosynthesis concepts and therapy aid clients’ personal and spiritual growth in a more defined religious environment, for example, within the pastoral counseling setting or as part of the curriculum of university students who are studying to become pastoral psychologists. Also, in this study, clients were unaware and not informed of psychosynthesis concepts and the psychosynthesis model of the human personality during their time in therapy. Future studies might, therefore, also conduct comparative research to discern whether an intellectual understanding of these concepts would influence the progress and/or degree of personal and spiritual growth.

## Conclusion

Throughout its history, the methods of the field of psychology have had “a shaky relationship with academia, and a particularly fraught and anxious connection to science” (Brottman 2011, p. xii). Pastoral psychologists have had an even greater challenge in their attempt to develop a psychological understand of spirituality within a scientific framework. In the words of the poet Tagore (1919), “A mind all logic is like a knife all blade. It makes the hand bleed that uses it” (p. 51). Perhaps more than ever before, pastoral psychologists need to resolve what Slife and Richardson (2014) called the “compatibility issue” by providing a bridge across the chasm between objective scientific methodology and the mysterious experiences of our lives; in other words, they need to find a way to stop the bleeding caused by purely logical thought. Ultimately, psychosynthesis embodies the idea that personal and spiritual growth entails a synthesis of the ineffable mysteries of the invisible with the reason and intellect of the visible. This article suggests that psychosynthesis provides a bridge between psychology and spirituality, indicating new ways to wisely hold the blade of logic so that, instead of wounding, it may discern what is the truth, the authentic, the sublime, and the higher potential held in all of life.
